# Supplementary figures and images for: The Use of Medicinal Plants in Blood Vessel Diseases: The Influence of Gender
Source: Life (Basel). 2023 Mar 23;13(4):866. doi: 10.3390/life13040866 (PMC10147070; doi:10.3390/life13040866)

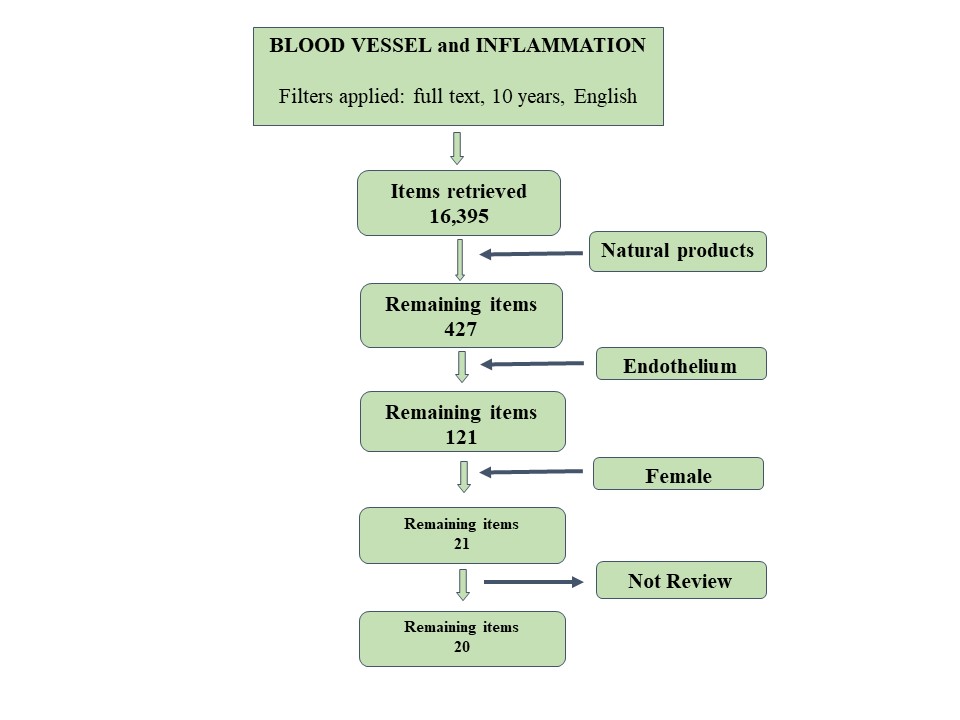

Supplement: Supplementary file 1 [file life-13-00866-s001.zip › life-2266574-supplementary.jpg]
